# Assessments of therapeutic effects according to timings for combined therapy with axitinib and immune check point inhibitor in a mouse renal cell carcinoma model

**DOI:** 10.1038/s41598-023-37857-9

**Published:** 2023-07-13

**Authors:** Hiromitsu Watanabe, Yuto Matsushita, Keita Tamura, Daisuke Motoyama, Takayuki Sugiyama, Atsushi Otsuka, Hideaki Miyake

**Affiliations:** Department of Urology, Hamamatsu University School of Medicine, 1-20-1 Handayama, Higashi-ku, Hamamatsu, 431-3192 Japan

**Keywords:** Cancer, Oncology, Urology

## Abstract

Recently, several types of systemic therapy using tyrosine kinase inhibitor (TKI) and immune checkpoint inhibitor (ICI) have been performed for advanced renal cell carcinoma (aRCC) patients; however, the optimal strategy of sequential treatment with these agents has not been well established. The objective of this study was to determine the differences of therapeutic effects according to timing for the introduction of TKI and ICI using a mouse RCC, RenCa model. The effects of combined treatment of TKI and/or ICI with axitinib, anti-mouse programmed death (PD)-1, or PD-ligand 1 (PD-L1) antibody on tumor growth and survival after subcutaneous and intravenous injection of RenCa cells, respectively, were compared according to three different treatment schedules: simultaneous administration, initial axitinib administration, and initial ICI administration. Infiltrating patterns of lymphocytes into tumors after combined treatments were evaluated by immunohistochemical staining. In mice treated with anti-PD-1 and anti-PD-L1 antibodies, significantly marked inhibitory effects on subcutaneous growth of tumors were observed in the simultaneous and initial ICI treatment groups, but not the group with the initial axitinib administration, compared to controls without treatment. Survival intervals of mice after intravenous injection of RenCa cells were significantly longer in the simultaneous and initial ICI administration, but not the initial axitinib administration, compared to the control. Furthermore, both CD8+ to CD3+ and CD8+ to CD11b+ T-lymphocyte ratios in subcutaneous RenCa tumors were significantly higher in the simultaneous and initial ICI administration, but not the initial axitinib administration, compared to the control. Favorable control against aRCC progression may be achieved by administering TKI and ICI simultaneously or ICI followed by TKI.

## Introduction

During the past 15 years, several types of systemic therapy, including molecular-targeted agents and immune checkpoint inhibitors (ICIs), have been introduced for the treatment of patients with advanced renal cell carcinoma (aRCC), based on promising outcomes in randomized clinical trials^1^. Of these, ICIs that target major molecules that mediate immune checkpoint pathways, such as programmed death-1 (PD-1), PD-ligand 1 (PD-L1), and cytotoxic T-lymphocyte antigen 4, currently play the central role in the sequential treatment of aRCC patients^2,3^. ICI-based combination therapy, consisting of either dual ICIs or ICI plus tyrosine kinase inhibitor (TKI), has been demonstrated to markedly improve prognostic outcomes of treatment-naïve aRCC patients. ICI was also shown to be effective in aRCC patients previously treated with TKIs^4,5^. In major clinical guidelines, ICI or ICI-based combination regimens are widely recommended as standard systemic therapies against aRCC^6,7^.

It has been well documented that pro-angiogenic factors promote tumor progression through modulations of immune microenvironment such as inhibition of dendric cell maturation, interference with the intratumoral infiltration of T-cells by disrupting tumor endothelium, and accumulation of immuno-suppressive cells, including myeloid-derived suppressor cells (MDSC), regulatory T cells, and tumor-associated macrophages^8^. Considering hypervascularity, one of the most prominent features of RCC, simultaneous treatment with TKI and ICI may exert additive or synergistic therapeutic effects on aRCC patients^9^. On the other hand, several studies reported durable response after discontinuation of ICIs in aRCC patients, which may reflect continuous effects of ICIs on intratumoral immune microenvironment; therefore, treatment with ICI followed by TKI may also show promising antitumor activity against aRCC^10,11^. To date, the optimal strategy of sequential treatment with ICI and TKI for aRCC patients has not been well characterized. Herein, we compared therapeutic effects of combined treatment with TKI and ICI using a mouse RCC model according to three different treatment schedules and investigated the infiltrating patterns of lymphocytes into tumor tissues after the completion of these treatments.

## Results

### Effects of treatment with axitinib and/or ICI on in vitro growth of RenCa

**Figure 1:**
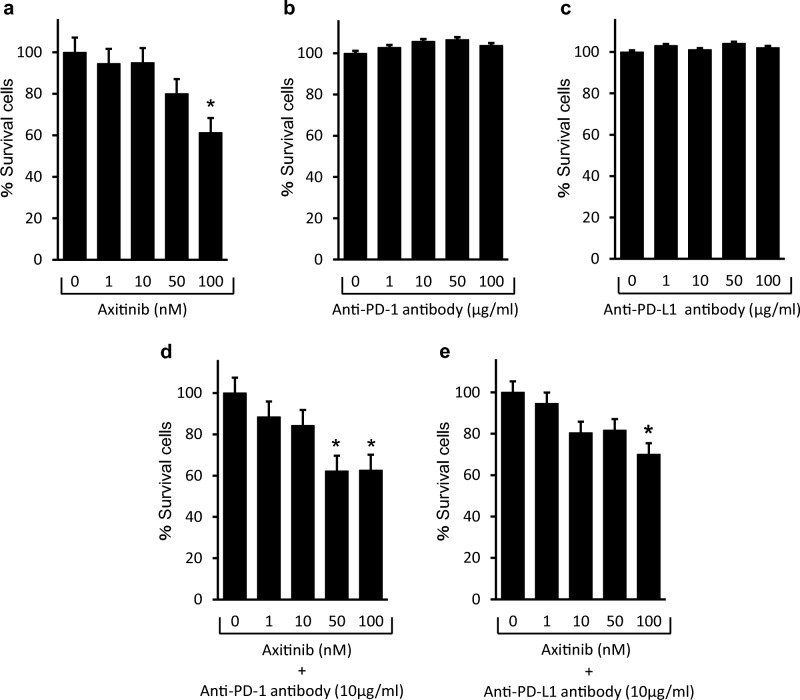
Effect of combined treatment with axitinib and/or immune checkpoint inhibitor on RenCa cell growth. RenCa cells were treated with axitinib (**a**), anti-mouse programmed death-1 (PD-1) antibody (**b**), anti-mouse PD-ligand 1 (PD-L1) antibody (**c**), axitinib plus 10 µg/ml anti-mouse PD-1 antibody (**d**) or axitinib plus 10 µg/ml anti-mouse PD-L1 antibody (**e**). After 72 h of incubation, the number of viable cells was determined by a cell proliferation assay. Each data point represents the mean of triplicate analyses; bars, standard deviations. Asterisk, differs from control (*p* < 0.01) by Student’s *t-*test.

As shown in Fig. 1a, axitinib inhibited the in vitro growth of RenCa cells in a dose-dependent manner; however, there was no significant effect of anti-mouse PD-1 or PD-L1 antibody on the in vitro growth of RenCa cells at any of the concentrations examined in this study (Fig. 1b,c). Furthermore, if combined with anti-mouse PD-1 or PD-L1 antibody, the sensitivity of RenCa cells to axitinib was not significantly enhanced (Fig. 1d, e).

### Effects of treatment with axitinib on expression levels of PD-L1 and PD-L2 in RenCa cells in vitro

**Figure 2:**
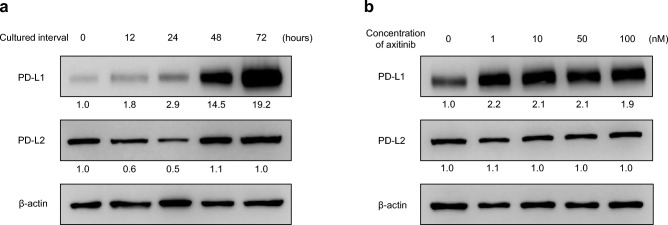
Effects of axitinib treatment on programmed death- ligand 1 (PD-L1) and PD-L2 expression in RenCa cells. Blots/gels in the figures have been cropped for clarity and conciseness. Original blots/gels are presented in Supplementary Fig. 1. (**a,b**) Use different blots/gels for respectively. (**a**) RenCa cells were treated with 50 nM axitinib for several intervals, and protein was then extracted from cultured cells and analyzed for PD-L1, PD-L2, and β-actin levels by Western blotting. These used the same membrane in all cases. Quantified expression levels of PD-L1 and PD-L2 relative to those of β-actin are presented below the corresponding point of Western blotting. (**b**) RenCa cells were treated with several concentrations of axitinib for 48 h, and protein was then extracted from cultured cells, and analyzed for PD-L1, PD-L2, and β-actin levels by Western blotting. These used the same membrane in all cases. Quantified expression levels of PD-L1 and PD-L2 relative to those of β-actin are presented below the corresponding point of Western blotting.

Western blot analyses were used to examine whether expression levels of PD-L1 and PD-L2 in RenCa cells were affected by treatment with axitinib. As shown in Fig. 2a, treatment of RenCa cells with axitinib induced a time-dependent up-regulation of PD-L1, but not of PD-L2. In addition, despite the lack of a dose-dependent effect, axitinib treatment resulted in increased PD-L1 expression in RenCa cells, while PD-L2 expression in RenCa cells was not affected by treatment with axitinib (Fig. 2b).

### Therapeutic effects of treatment with axitinib, ICI, or its combination in mice receiving subcutaneous or intravenous injection of RenCa cells

**Figure 3:**
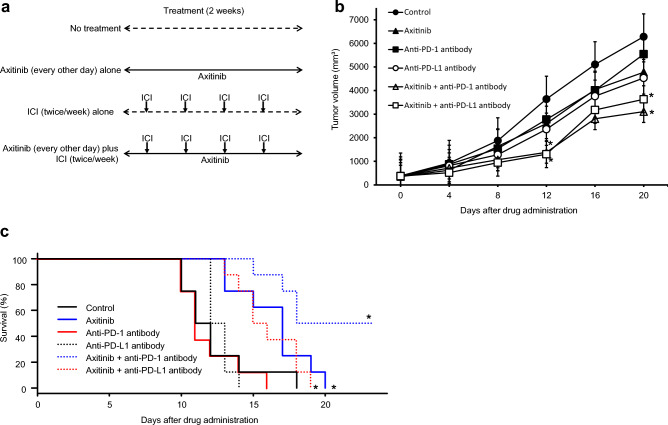
Therapeutic effects of combined treatment with axitinib, immune checkpoint inhibitor (ICI), or its combination on in vivo RenCa models. (**a**) Schematic presentation of treatment schedules in this experiment. (**b**) One week after subcutaneous injection of RenCa cells, mice bore approximately 100 mm^3^ of tumors, and were randomly selected for the following six groups: no treatment, axitinib alone, anti-mouse programmed death-1 (PD-1) antibody alone, anti-mouse PD-ligand 1 (PD-L1) antibody alone, axitinib plus anti-mouse PD-1 antibody, and axitinib plus anti-mouse PD-L1 antibody. Detailed treatment schedules using axitinib and/or ICIs are described in the “Materials and methods” section. Each data point represents the mean tumor volume in each experimental group (n = 8 mice); bars, standard deviation. Mean tumor volumes 21 days after starting treatment relative to that of no treatment group were as follows: −23.9%, axitinib alone; −11.8%, anti-mouse PD-1 antibody alone; −27.8%, anti-mouse PD-L1 antibody; −50.6%, axitinib plus anti-mouse PD-1 antibody; and −42.2%, axitinib plus anti-mouse PD-L1 antibody. Asterisk, differs from controls (*p* < 0.05) by Student’s *t-*test. (**c**) Mice after intravenous injection of RenCa cells were randomly selected for the six groups as described above. Detailed treatment schedules using axitinib and/or ICIs are described in the “Materials and methods” section. Each survival curve represents the changes in survival rate in each experimental group (n = 8 mice). Asterisk, differs from controls (*p* < 0.05) by log-rank test.

Although treatment with axitinib, anti-mouse PD-1 antibody, or PD-L1 antibody inhibited the growth of subcutaneous tumors, there was no significant difference in subcutaneous tumor volumes between mice without any treatment and those receiving single agents. However, tumor volumes in mice after combined treatments with axitinib plus anti-mouse PD-1 antibody and axitinib plus anti-mouse PD-L1 antibody were significantly smaller than those in mice without any treatment (Fig. 3b).

In mice given intravenous injection of RenCa cells, treatment with anti-mouse PD-1 antibody or PD-L1 antibody did not improve survival; however, the survival of mice treated with axitinib, axitinib plus anti-mouse PD-1 antibody, and axitinib plus anti-mouse PD-L1 antibody were significantly longer than those of mice without treatment (Fig. 3c).

### Therapeutic effects of sequential treatment orders with axitinib and ICI in mice receiving subcutaneous or intravenous injection of RenCa cells

**Figure 4:**
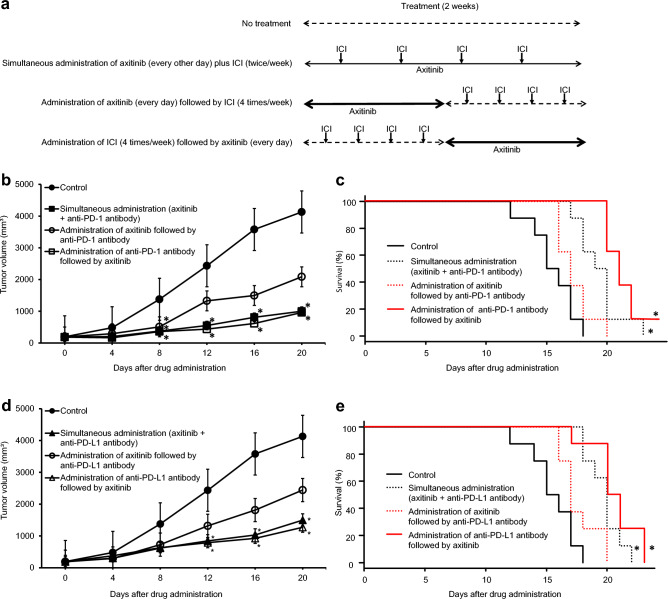
Therapeutic effects of three different sequential treatment orders with axitinib and immune checkpoint inhibitor (ICI) on in vivo RenCa models. (**a**) Schematic presentation of treatment schedules in this experiment. (**b**) One week after subcutaneous injection of RenCa cells, mice bore approximately 100 mm^3^ of tumors, and were randomly selected for the following four groups: no treatment, simultaneous administration of axitinib plus anti-mouse programmed death-1 (PD-1) antibody, administration of axitinib followed by anti-mouse PD-1 antibody, and administration of anti-mouse PD-1 antibody followed by axitinib. Detailed treatment schedules using axitinib and anti-mouse PD-1 antibody are described in the “Materials and methods” section. Each data point represents the mean tumor volume in each experimental group (n = 8 mice); bars, standard deviation. Mean tumor volumes 21 days after starting treatment relative to that of no treatment group were as follows: −75.8%, simultaneous administration of axitinib plus anti-mouse PD-1 antibody; −49.5%, administration of axitinib followed by anti-mouse PD-1 antibody; and −76.7%, administration of anti-mouse PD-1 antibody followed by axitinib. Asterisk, differs from controls (*p* < 0.05) by Student’s *t-*test. (**c**) Mice after intravenous injection of RenCa cells were randomly selected for the four groups as described above. Detailed treatment schedules using axitinib and anti-mouse PD-1 antibody are described in the “Materials and methods” section. Each survival curve represents the changes in survival rate in each experimental group (n = 8 mice). Asterisk, differs from controls (*p* < 0.05) by log-rank test. (**d**) One week after subcutaneous injection of RenCa cells, mice bore approximately 100 mm^3^ of tumors, and were randomly selected for the following four groups: no treatment, simultaneous administration of axitinib plus anti-mouse programmed death-ligand 1 (PD-L1) antibody, administration of axitinib followed by anti-mouse PD-L1 antibody, and administration of anti-mouse PD-L1 antibody followed by axitinib. Detailed treatment schedules using axitinib and anti-mouse PD-L1 antibody are described in the “Materials and methods” section. Each data point represents the mean tumor volume in each experimental group (n = 8 mice); bars, standard deviation. Mean tumor volumes 21 days after starting treatment relative to that of no treatment group were as follows: −63.7%, simultaneous administration of axitinib plus anti-mouse PD-L1 antibody; −40.9%, administration of axitinib followed by anti-mouse PD-L1 antibody; and −69.2%, administration of anti-mouse PD-L1 antibody followed by axitinib. Asterisk, differs from controls (*p* < 0.05) by Student’s *t-*test. (**e**) Mice after intravenous injection of RenCa cells were randomly selected for the four groups as described above. Detailed treatment schedules using axitinib and anti-mouse PD-L1 antibody are described in the Materials and Methods section. Each survival curve represents the changes in survival rate in each experimental group (n = 8 mice). Asterisk, differs from controls (*p* < 0.05) by log-rank test.

There was no significant difference in the subcutaneous tumor volumes between control mice without treatment and those receiving axitinib followed by anti-mouse PD-1 antibody; however, tumor volumes in mice simultaneously receiving axitinib plus anti-mouse PD-1 antibody and those receiving anti-mouse PD-1 antibody followed by axitinib were significantly lower than those in mice without treatment (Fig. 4b). Moreover, despite the lack of significant difference in survival between the control group without treatment and the initial axitinib administration after intravenous injection of RenCa cells, survival in the simultaneous administration and the initial anti-mouse PD-1 antibody administration were significantly prolonged compared to the control (Fig. 4c).

Similarly, significantly marked inhibitory effects on subcutaneous tumor growth were detected in the simultaneous administration and initial anti-mouse PD-L1 antibody administration, but not the initial axitinib administration, compared to those in the control without treatment (Fig. 4d). Furthermore, survival of mice after intravenous injection of RenCa cells were significantly longer in the simultaneous administration and initial anti-mouse PD-L1 antibody administration, but not the initial axitinib administration, than in the control (Fig. 4e).

### Assessments of infiltrating patterns of T-lymphocytes into subcutaneous RenCa tumors after combined treatments

**Figure 5:**
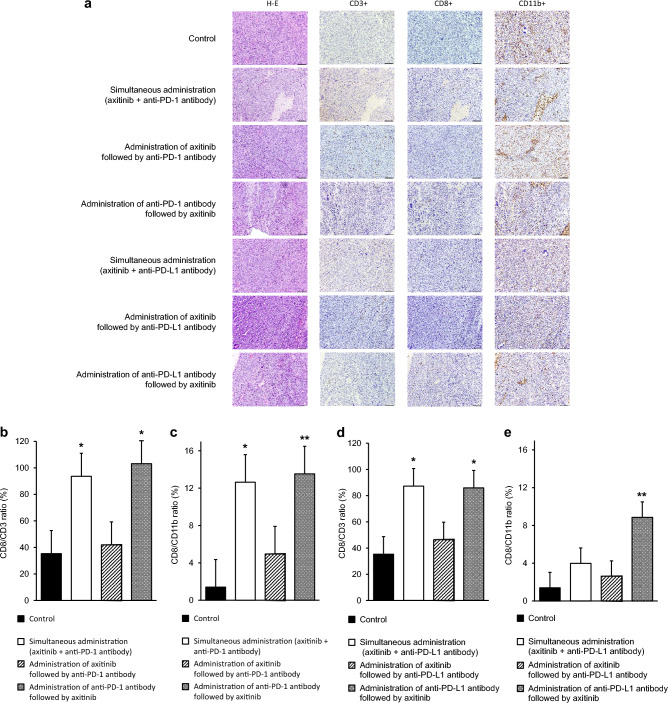
Histopathological study of RenCa tumors after three different sequential treatment orders with axitinib and immune checkpoint inhibitor (ICI). In vivo subcutaneous tumors were harvested from nude mice after completing treatment according to the schedule shown in this figure. Sections from each tumor tissue were examined by hematoxylin–eosin (H–E) staining and immunohistochemical staining with antibodies against CD3, CD8, and CD11b. (**a**) Representative findings of histopathological study are shown (scale bar = 100 µm). (**b**) Quantification of CD8/CD3 ratio in RenCa tumors based on the findings of immunohistochemical staining according to the following treatment group: no treatment, simultaneous administration of axitinib plus anti-mouse programmed death-1 (PD-1) antibody, administration of axitinib followed by anti-mouse PD-1 antibody, and administration of anti-mouse PD-1 antibody followed by axitinib. Each data point represents the mean of eight tumors; bars, standard deviations (SDs). Asterisk, differs from control (p < 0.05) by Student’s *t-*test. (**c**) Quantification of CD8/CD11b ratio in RenCa tumors based on the findings of immunohistochemical staining according to the treatment group described in (**b**). Each data point represents the mean of eight tumors; bars, SDs. Asterisk and double asterisk, differ from control (*p* < 0.05 and < 0.01, respectively) by Student’s *t-*test. (**d**) Quantification of CD8/CD3 ratio in RenCa tumors based on the findings of immunohistochemical staining according to the following treatment groups: no treatment, simultaneous administration of axitinib plus anti-mouse programmed death-ligand 1 (PD-L1) antibody, administration of axitinib followed by anti-mouse PD-L1 antibody, and administration of anti-mouse PD-L1 antibody followed by axitinib. Each data point represents the mean of eight tumors; bars, SDs. Asterisk, differs from control (*p* < 0.05) by Student’s *t-*test. (**e**) Quantification of CD8/CD11b ratio in RenCa tumors based on the findings of immunohistochemical staining according to the treatment group described in (**d**). Each data point represents the mean of eight tumors; bars, SDs. Asterisk, differs from control (*p* < 0.01) by Student’s *t-*test.

Infiltrations of CD3+, CD8+, and CD11b+ T-lymphocytes into subcutaneous tumors after combined treatment with axitinib and ICI were quantitatively evaluated based on the outcomes of immunohistochemical staining. Figure 5a presents the typical findings on immunohistochemical staining of subcutaneous tumors. As shown in Fig. 5b, c, both CD8+ to CD3+ (CD8/CD3) and CD8+ to CD11b+ (CD8/CD11b) T-lymphocyte ratios in subcutaneous tumors were significantly higher in the simultaneous administration and initial anti-mouse PD-1 antibody administration, but not the initial axitinib administration, than in the control without treatment. In addition, CD8/CD3 ratio in subcutaneous tumors were significantly higher in the simultaneous administration and initial anti-mouse PD-L1 antibody administration, but not the initial axitinib administration, than in the control, while only CD8/CD11b ratio in the initial anti-mouse PD-L1 antibody administration was significantly higher than in the control (Fig. 5d, e).

## Discussion

In recent years, a wide variety of novel agents with different mechanisms of action have been introduced in routine clinical practice for treatment of patients with aRCC, and there have been marked changes in the therapeutic strategy against aRCC^12^. Due to several advantageous characteristics, ICIs play a central role in systemic therapy for aRCC patients, irrespective of risk classification or histopathology^13^. In particular, ICI-based combination therapy, consisting of either dual use of ICIs or ICI plus TKI, is regarded as a standard of care, and is widely applied to treatment-naïve aRCC patients^1,7^. However, whether the efficacy of ICI-based combination therapy is modulated by its order of treatment is unclear. In this study, we compared the therapeutic effects of three different sequential treatment orders with TKI and ICI using a mouse RCC, RenCa model.

Considering the main objective of this study to investigate in vivo effects of several treatments, including ICIs, and the mechanisms mediating their antitumor activities, human RCC cell lines could not be suitable; thus, RenCa, the most frequently used mouse RCC cell lines, was selected in this study. We initially confirmed that treatment with axitinib resulted in a dose-dependent growth inhibition of RenCa cells in vitro, while in vitro growth of RenCa cells was not affected by treatment with anti-PD-1 or PD-L1 antibody, either alone or in combination with axitinib. Furthermore, after treatment of RenCa cells with axitinib in vitro, despite the lack of marked changes in PD-L2 expression, PD-L1 was shown to be significantly up-regulated in a time-dependent manner. Similar to in vitro studies, increased expression of PD-L1, but not PD-L2, was confirmed after administration of axitinib in vivo (Supplementary Fig. 2). In a previous study by Allen et al., the up-regulation of an adaptive immunosuppressive pathway, including PD-L1, during antiangiogenic therapy was demonstrated in several preclinical models^14^. To date, there have been a number of studies characterizing the important role of PD-L1 in enhancing malignant potential of RCC. Wang et al. reported the induction of epithelial-mesenchymal transition by PD-L1, resulting in the enhancement of stemness in RCC^15^. Collectively, these findings suggest strong rationale to combine ICI with axitinib as a systemic approach for aRCC.

In the in vivo experiments of this study, efficacies of TKI, ICI, and its combination were compared in two models using mice given subcutaneous and intravenous injection with RenCa cells. As expected, combined treatment with axitinib plus ICI, irrespective of anti-PD-1 or PD-L1 antibody, exhibited therapeutic effects superior to treatment with either agent alone in terms of tumor growth inhibition and prolonged survival in mice after subcutaneous and intravenous injection of RenCa cells, respectively. Taken together, these two in vivo RenCa models may be reliable tools for the assessment of anti-tumor activities of experimental therapies against aRCC.

In subsequent in vivo studies, therapeutic effects of three different schedules of sequential combination with axitinib and ICI were evaluated using two types of RenCa model. Regardless of anti-PD-1 or PD-L1 antibody levels, simultaneous treatment with axitinib plus ICI and preceding treatment with ICI followed by axitinib were shown to achieve favorable results regarding both tumor growth inhibition and prolonged survival compared to preceding treatment with axitinib followed by ICI. Further studies are needed to explore the mechanism underlying these outcomes. One of the most convincing mechanisms is durable response by ICI, even after its discontinuation, which has been reported to be frequently observed in aRCC patients treated with ICI^11,16^. In addition, preceding treatment with standard dose of axitinib alone under the absence of ICI-induced effects could negatively impact the tumor microenvironment, leading to "re-hypoxia", which induces infiltration of immune-suppressive cells and up-regulation of PD-L1^17,18^.

Another point of interest is changes in infiltration patterns of tumor-infiltrating lymphocytes (TIL) after three different schedules of sequential combination with axitinib and ICI. Although the significance of TIL subsets after ICI-based systemic therapy has not been well documented, a number of previous studies have examined the presence of several TILs in tumor tissues prior to systemic therapy, and proportions of specific TILs were shown to be associated with prognostic outcomes^19,20^. Peng et al. reported that high CD8/CD3 ratio in liver oligometastasis from colorectal cancer was independently correlated with better recurrence-free and overall survivals^19^. In the present study, both CD8/CD3 and CD8/CD11b ratios in subcutaneous tumors closely reflected efficacies of different sequential treatment orders with axitinib and ICI. Accordingly, assessments of TIL subsets during ICI-based therapies may be useful as both predictive and prognostic biomarkers for aRCC.

This study had several limitations. First, RenCa model was used throughout this study. In addition, one of the most efficacious TKIs, axitinib alone, was selected for the experiments of this study; thus, it will be necessary to confirm whether the present findings could be reproducible when using TKIs other than axitinib and other tumor-derived animal models. Second, in the assessment of TIL subsets, infiltration of CD11b+ T lymphocytes were quantified based on the findings of immunohistochemical staining; however, it would be preferable to evaluate MDSC rather than CD11b+ T lymphocyte. Third, baseline mean tumor volumes before treatment were not identical among animal studies performed in this study, and more marked antitumor effects were observed in mice bearing small tumors than in those large tumors; therefore, it should consider this point, when interpreting the results of these experiments. Fourth, although one of the key aims of combined treatment involving ICI is to achieve long-term eradication of tumors, any regimens examined in this study failed to show complete response. Therefore, it is necessary to investigate more effective sequential therapies in animal studies, that enable to completely eradicate tumors, at least in a proportion of mice. Finally, if the present findings were to be used in clinical practice, it would be expected to alleviate severe adverse events associated with simultaneous administration of TKI and ICI by preceding administration of ICI followed by TKI; thus, further studies to investigate safety profiles among three different schedules of sequential combination with TKI and ICI are needed.

## Conclusion

Simultaneous treatment with axitinib plus ICI or preceding treatment with ICI followed by axitinib efficaciously inhibited tumor growth and prolonged survival in mice after subcutaneous and intravenous injection of RenCa cells, respectively, and these therapeutic effects were proportional to post-treatment CD8/CD3 and CD8/CD11b ratios in subcutaneous tumors. Collectively, these findings suggest that it may be expected to achieve therapeutic effects of preceding treatment with ICI followed by TKI on aRCC similar to those of simultaneous treatment with TKI and ICI.

## Materials and methods

### Tumor cell line

RenCa, a mouse RCC of BALB/c origin, was purchased from JCRB cell bank (Osaka, Japan), and was cultured with in RPMI 1640 supplemented with 10% fetal bovine serum, 100 IU/mL penicillin, and 100 µg/mL streptomycin.

### Cell proliferation assay

In vitro growth inhibitory effects of TKI and/or ICI on RenCa cells were assessed using Cell Counting Kit-8 (Dojindo Laboratories, Kumamoto, Japan). Briefly, 5 × 10^3^ cells were seeded in each well of 96-well plates and allowed to attach overnight. The wells were treated with vehicle, axitinib (Pfizer, Inc., New York, NY, USA), anti-mouse PD-1 antibody (Bio X Cell, Lebanon, NH, USA), anti-mouse PD-L1 antibody (Bio X Cell), axitinib plus anti-mouse PD-1 antibody, or axitinib plus anti-mouse PD-L1 antibody. After 72 h of incubation, the number of cells was counted. Each assay was performed in triplicate.

### Western blot analysis

Western blot analysis was performed as previously described^21^. Briefly, samples containing equal amounts of protein (20 µg) from lysates of RenCa cells after treatment with axitinib were subjected to sodium dodecyl sulfate–polyacrylamide gel electrophoresis and transferred to a nitrocellulose filter. The filter was blocked in phosphate-buffered saline containing 5% nonfat milk powder at 4 °C overnight and then incubated for 1 h with antibody against mouse PD-L1, PD-L2 (Abcam, Cambridge, UK), or β-actin (Cell Signaling Technology, Danvers, MA, USA). The filters were then incubated for 30 min with horseradish peroxide-conjugated secondary antibody (Cell Signaling Technology), and specific proteins were detected using an enhanced chemiluminescence Western Blot analysis system (GE Healthcare Japan, Tokyo, Japan). The strength of each signal density was determined using a densitometer (Bio-Tek Instruments Inc., Winooski, VT, USA).

### Animal studies

Eight week-old-male, specific pathogen-free, Balb/c mice were purchased from Japan SLC (Shizuoka, Japan), and housed in a controlled environment at 22 °C on a 12-h light, 12-h dark cycle. All animal experiments were performed in accordance with the National Institutes of Health Guide for the Care and Use of Laboratory Animals. Approximately 5 × 10^6^ of RenCa cells were injected either subcutaneously with 100 µl of Matrigel (CORNING, Corning, NY, USA) into the left flank or intravenously into the tail vein. One weeks after the injection of RenCa cells, treatment of mice was started as described below. Mice after subcutaneous injection of RenCa cells were daily observed for 21 days after starting treatment, and then sacrificed, while mice after intravenous injection of RenCa cells were daily observed for 24 days or until death. Considering cachexic appearance, mice were euthanized approximately three weeks after the initiation of treatment by cervical dislocation. When mice never breathed after dislocation, euthanasia was judged to be successful. Each experimental group consisted of eight mice. The required sample size was determined based on the report of Charan et al.^22^. Twenty-four groups of experiments were conducted and a total of 192 mice were used.

In the first experiment, therapeutic effects of ICI, TKI, and its combination were examined using both mice receiving either subcutaneous or intravenous injection of RenCa cells. Mice were randomly divided into the following six groups: no treatment, axitinib alone, anti-mouse PD-1 antibody alone, anti-mouse PD-L1 antibody alone, axitinib plus anti-mouse PD-1 antibody, and axitinib plus anti-mouse PD-L1 antibody. After randomization, axitinib at a dose of 25 mg/kg was administered orally every other day for 2 weeks, and 200 µg of anti-mouse PD-1 antibody or anti-mouse PD-L1 antibody was injected intraperitoneally twice per week for 2 weeks.

In the second experiment, efficacies of three different sequential treatment orders with TKI and ICI were compared using both subcutaneous and intravenous injection models. Mice were randomly selected for the following seven treatments: no treatment, simultaneous administration of axitinib plus anti-mouse PD-1 antibody, administration of axitinib followed by anti-mouse PD-1 antibody, administration of anti-mouse PD-1 antibody followed by axitinib, simultaneous administration of axitinib plus anti-mouse PD-L1 antibody, administration of axitinib followed by anti-mouse PD-L1 antibody, and administration of anti-mouse PD-L1 antibody followed by axitinib. After randomization, axitinib at a dose of 25 mg/kg was administered orally every other day for 2 weeks and 200 µg of anti-mouse PD-1 antibody or anti-mouse PD-L1 antibody was injected intraperitoneally twice per week for 2 weeks in the simultaneous treatment group, axitinib at a dose of 25 mg/kg was daily administered orally for the first 1 week followed by the intraperitoneal injection of 200 µg of anti-mouse PD-1 antibody or anti-mouse PD-L1 antibody four times per week for the next 1 week in the axitinib-preceding group, and 200 µg of anti-mouse PD-1 antibody or anti-mouse PD-L1 antibody was injected intraperitoneally four times per week for the first 1 week followed by the daily oral administration of axitinib at a dose of 25 mg/kg for the next 1 week in the ICI-preceding group.

Mice after subcutaneous injection of RenCa cells were daily observed for 21 days after starting treatment and then sacrificed, while mice after intravenous injection were daily observed for 24 days or until death. The growth of subcutaneous tumor was measured twice per week using calipers and the tumor volume was calculated according to the formula: 1/2 × (shortest diameter)^2^ × (the longest diameter). Three weeks after starting treatment, all living mice were sacrificed, and tumor tissues and lungs were harvested from mice receiving subcutaneous and intravenous tumor cell injection, respectively. We alternated treatment group assignment, treatment, and measurements with experimental assistants and other researcher to minimize randomization and potential confounders. All animal experiments were approved by the Animal Care and Use Committee of Hamamatsu University School of Medicine (approved number: 2020030) and were performed according to the guidelines of this committee and ARRIVE^23^.

### Immunohistochemical staining

Subcutaneous tumors were harvested from mice after the completion of treatment with TKI and/or ICI as described above. Immunohistochemical staining of tumor specimens was performed as reported previously^24^. Briefly, sections from formaldehyde-fixed, paraffin-embedded tissue were deparaffinized with xylene and rehydrated in a graded series of alcohol and distilled water. After endogenous peroxidase was blocked with 3% hydrogen peroxide in distilled water, sections were stained with antibodies against mouse CD3, CD8 (Cell Signaling Technology), and CD11b (Abcam) at 4 °C overnight. Sections were then reacted with horseradish peroxidase-conjugated anti-Rabbit IgG secondary antibody (Cell-signaling Technology) for 30 min at room temperature. After incubation in avidin–biotin peroxidase complex for 30 min at room temperature, samples were exposed to diaminobenzidine tetrahydrochloride solution and counterstained using hematoxylin.

Quantitative assessments of the findings of immunohistochemical staining were performed using Fiji software as previously described^25^. Five sections per tumor were prepared in the immediate vicinity of each tissue, and the number of positive cells after immunohistochemical staining were quantified at five random locations per section.

### Statistical analysis

All statistical analyses were performed using EZR software (Saitama Medical Center, Jichi Medical University, ver. 1.40), and *p* values less than 0.05 were considered significant. All data are presented as mean ± standard deviation, and differences between the two groups were compared using the two-tailed Student *t*-test. Survival rates of mice after starting treatment were calculated by the Kaplan–Meier method, and differences were analyzed by the log-rank test.

### Ethical approval

All applicable international, national, and/or institutional guidelines for the care and use of animals were followed. The study was approved by a Research Ethics Committee at the institution.

## Supplementary Information

Supplementary Figure 1.

Supplementary Figure 2.

## Data Availability

The datasets used and analyzed during the current study available from the corresponding author on reasonable request.
