# Supplementary material for: Assessments of therapeutic effects according to timings for combined therapy with axitinib and immune check point inhibitor in a mouse renal cell carcinoma model
Source: Sci Rep. 2023 Jul 13;13:11361. doi: 10.1038/s41598-023-37857-9 (PMC10344912; doi:10.1038/s41598-023-37857-9)

**Assessments of optimal timing for combined therapy with axitinib and immune check point inhibitor in a mouse renal cell carcinoma model**

Hiromitsu Watanabe,* Yuto Matsushita, Keita Tamura, Daisuke Motoyama, Takayuki Sugiyama, Atsushi Otsuka, Hideaki Miyake

*Department of Urology, Hamamatsu University School of Medicine, Hamamatsu, Japan*

Running head: Optimal timing for combined therapy with axitinib and immune check point inhibitor

**Corresponding author:* Address: Department of Urology, Hamamatsu University School of Medicine, 1-20-1 Handayama, Higashi-Ku, Hamamatsu 431-3192, Japan. Tel.: +81 534352306; Fax: +81 534352305.

*E-mail address:* urohiro@hama-med.ac.jp

**Supplementary Figure 1.** Effects of axitinib treatment on programmed death- ligand 1 (PD-L1) and PD-L2 expression in RenCa cells. Original blots/gels are presented. “a (Cultured interval)” and “b (Concentration of axitinib)” use different blots/gels for respectively.


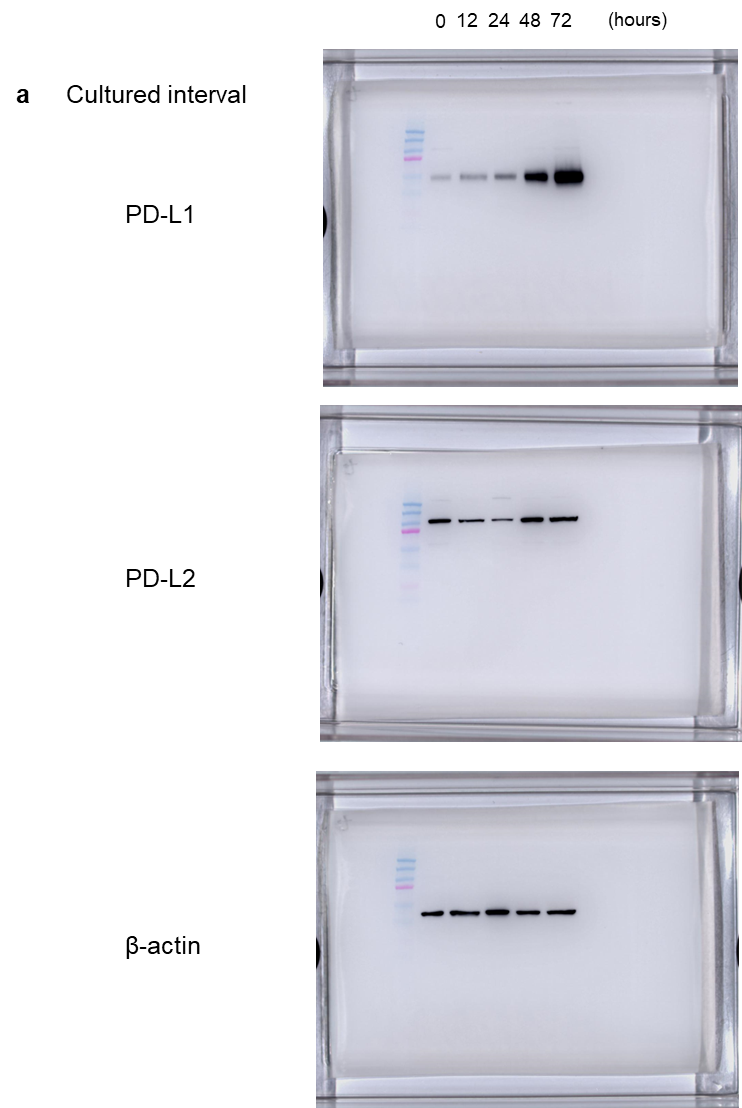


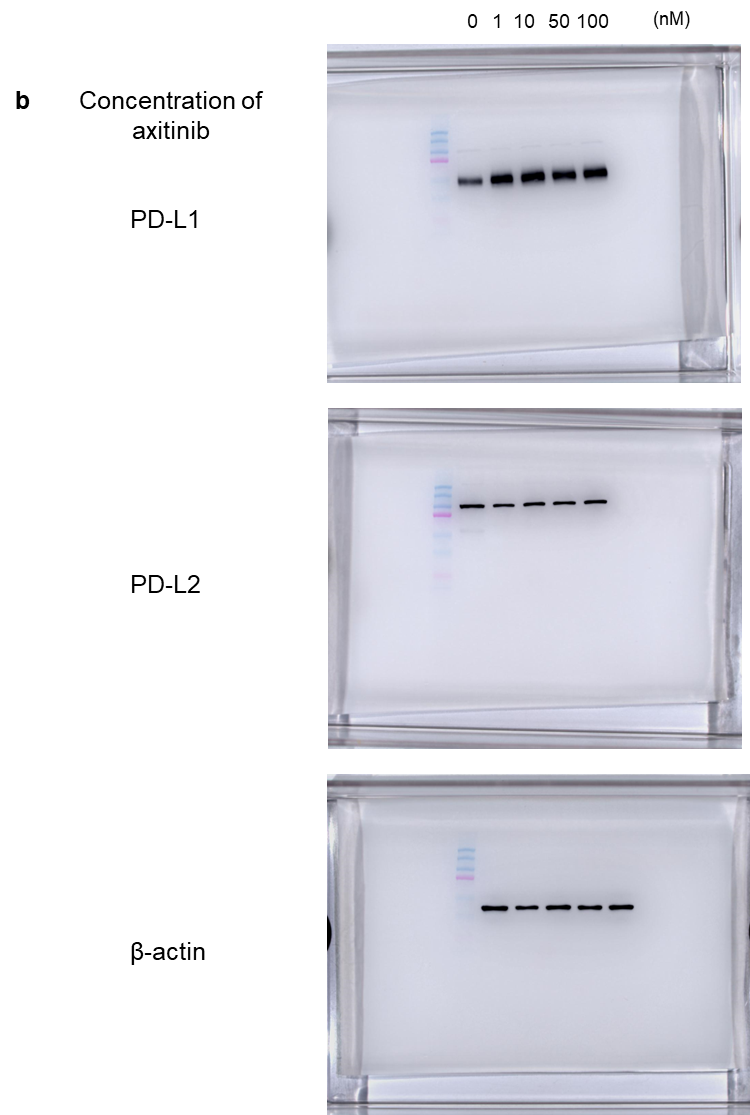

Supplement: Supplementary file 1 — Supplementary Figure 1. [file 41598_2023_37857_MOESM1_ESM.docx]
