# Supplementary figures and images for: Assessments of therapeutic effects according to timings for combined therapy with axitinib and immune check point inhibitor in a mouse renal cell carcinoma model
Source: Sci Rep. 2023 Jul 13;13:11361. doi: 10.1038/s41598-023-37857-9 (PMC10344912; doi:10.1038/s41598-023-37857-9)

Supplementary Figure 2 :Proportion of cells expressing PD-L1 / PD-L2 *in vivo*

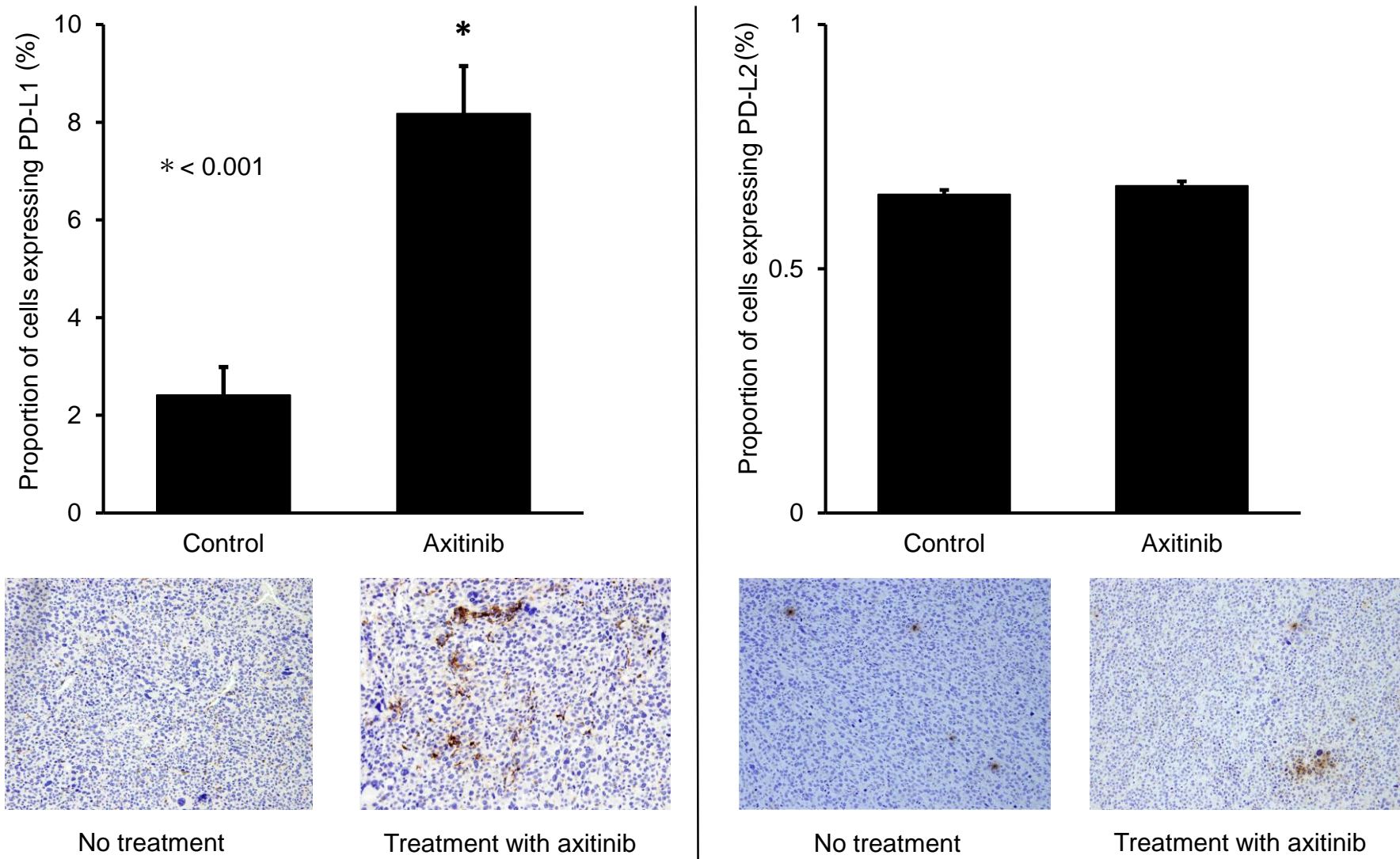

Supplement: Supplementary file 2 — Supplementary Figure 2. [file 41598_2023_37857_MOESM2_ESM.pdf]
